# Allogeneic Hematopoietic Cell Transplantation in Advanced Systemic Mastocytosis: A retrospective analysis of the DRST and GREM registries

**DOI:** 10.1038/s41375-024-02186-x

**Published:** 2024-03-06

**Authors:** Johannes Lübke, Deborah Christen, Juliana Schwaab, Anne Kaiser, Nicole Naumann, Khalid Shoumariyeh, Madlen Jentzsch, Katja Sockel, Judith Schaffrath, Francis A. Ayuk, Matthias Stelljes, Inken Hilgendorf, Elisa Sala, Jennifer Kaivers, Stefan Schönland, Christoph Wittke, Bernd Hertenstein, Markus Radsak, Ulrich Kaiser, Valeska Brückl, Nicolaus Kröger, Tim H. Brümmendorf, Wolf-Karsten Hofmann, Stefan Klein, Edgar Jost, Andreas Reiter, Jens Panse

**Affiliations:** 1 Department of Hematology and Oncology, University Hospital Mannheim, Heidelberg University, Mannheim, Germany; 2 Department of Oncology, Hematology, Hemostaseology and Stem Cell Transplantation, University Hospital RWTH Aachen, Aachen, Germany & Center for Integrated Oncology (CIO), Aachen, Bonn, Cologne, Düsseldorf (ABCD), Aachen, Germany; 3 Department of Medicine I, Medical Center- University of Freiburg, Faculty of Medicine, University of Freiburg, Freiburg, Germany and German Cancer Consortium (DKTK), Partner Site Freiburg, Freiburg, Germany; 4 Clinic and Policlinic for Hematology and Cellular Therapy, University of Leipzig Medical Center, Leipzig, Germany; 5 Medical Department I, University Hospital Carl Gustav Carus, TU Dresden, Dresden, Germany; 6 Department of Internal Medicine IV, Hematology and Oncology, Martin Luther University Halle-Wittenberg, Halle (Saale), Germany; 7 Department of Stem Cell Transplantation with Research Department Cell and Gene Therapy University Medical Center Hamburg-Eppendorf, Hamburg, Germany; 8 Department of Medicine A/Hematology and Oncology, University of Muenster, Münster, Germany; 9 Universitätsklinikum Jena, Klinik für Innere Medizin II, Abteilung für Hämatologie und Internistische Onkologie, Jena, Germany; 10 Department of Internal Medicine III, University Hospital Ulm, Ulm, Germany; 11 Department of Hematology and Stem Cell Transplantation, West German Cancer Center, University Hospital Essen, University of Duisburg-Essen, Essen, Germany; 12 Department of Internal Medicine V, Division of Hematology/Oncology, Heidelberg University Hospital, Heidelberg, Germany; 13 Department of Medicine, Clinic III, Hematology, Oncology and Palliative Medicine, Rostock University Medical Center, Rostock, Germany; 14 Klinikum Bremen-Mitte, Bremen, Germany; 15 3rd Department of Medicine, Johannes Gutenberg University Medical Center, Mainz, Germany; 16 Department of Internal Medicine III, University Hospital Regensburg, Regensburg, Germany; 17 Department of Internal Medicine 5, Hematology and Oncology, University Hospital Erlangen, Erlangen, Germany

**Keywords:** Translational research, Myeloproliferative disease

## Abstract

We identified 71 patients with AdvSM (aggressive SM [ASM], SM with an associated hematologic neoplasm [SM-AHN, e.g., acute myeloid leukemia, SM-AML], mast cell leukemia [MCL]) in two national registries (DRST/GREM) who received an allogeneic hematopoietic cell transplantation (alloHCT) performed in Germany from 1999–2021. Median overall survival (OS) of ASM/SM-AHN (*n* = 30, 45%), SM-AML (*n* = 28, 39%) and MCL ± AHN (*n* = 13, 19%) was 9.0, 3.3 and 0.9 years (*P* = 0.007). Improved median OS was associated with response of SM (17/41, 41%; HR 0.4 [0.2–0.9], *P* = 0.035) and/or of AHN (26/43, 60%, HR 0.3 [0.1–0.7], *P* = 0.004) prior to alloHCT. Adverse predictors for OS included absence of *KIT* D816V (10/61, 16%, HR 2.9 [1.2–6.5], *P* < 0.001) and a complex karyotype (9/60, 15%, HR 4.2 [1.8–10.0], *P* = 0.016). HLA-match, conditioning type or transplantation at centers reporting above-average alloHCTs (≥7) had no impact on OS. Taking into account competing events at years 1, 3 and 5, relapse-related mortality and non-relapse mortality rate were 15%/23%, 20%/30% and 23%/35%, respectively. Irrespective of subtype, subsequent treatment response was achieved in 13/30 (43%) patients and was highest on midostaurin/avapritinib (7/9, 78%). We conclude that outcome of alloHCT in AdvSM is more affected by disease phenotype and treatment response prior to transplant than by transplant characteristics.

## Introduction

Systemic mastocytosis (SM) is characterized by accumulation of clonal, neoplastic mast cells within the bone marrow and additional organ systems, e.g., skin and gastrointestinal tract [1, 2]. Advanced systemic mastocytosis (AdvSM) comprises aggressive systemic mastocytosis (ASM), SM with an associated hematologic or myeloid neoplasm (SM-AHN/SM-AMN) according to World Health Classification (WHO)-HAEM5 or International Consensus Classification (ICC), respectively, and mast cell leukemia (MCL) ± AHN as the most aggressive subtype impacting on overall survival (OS) [3, 4]. In >90% of patients, a somatic point mutation in *KIT* at codon 816 (*KIT* D816V) is the primary disease driver [5–7]. In AdvSM, 60–80% of patients harbor additional somatic mutations, some of which confer an adverse impact on prognosis, e.g., mutations in *SRSF2, ASXL1, RUNX1* (*S/A/R* gene panel), *EZH2*, *JAK2*, and others [8–12].

In addition to conventional chemotherapy, e.g. to the purine analog cladribine [13–16], the recent development of targeted treatments, e.g. with the multikinase/KIT inhibitor midostaurin [17–21] or the specific KIT D816V inhibitor avapritinib, has substantially extended therapeutic alternatives [22–25]. However, with data from only one larger retrospective analysis on 57 patients available, timing, type of conditioning and post-transplant strategies of allogeneic hematopoietic cell transplantation (alloHCT) as the only curative treatment option remain elusive so far [26]. The advent of reduced intensity conditioning (RIC) [27] and alternate donor sources (e.g. haploidentical alloHCT) [28, 29], have expanded the availability of alloHCT to an increasing number of patients. However, outcome of alloHCT is affected by relapse and non-relapse morbidity and mortality (NRM).

We therefore sought to retrospectively analyze the impact of baseline characteristics, response status and various transplant settings on response, progression-free (PFS) and overall survival (OS) after alloHCT in patients with diagnosis of AdvSM and enrollment within the ‘German Stem Cell Transplantation Registry’ (DRST) or the ‘German Registry on Eosinophils and Mast Cells’ (GREM).

## Methods

### Data collection

**Fig. 1:**
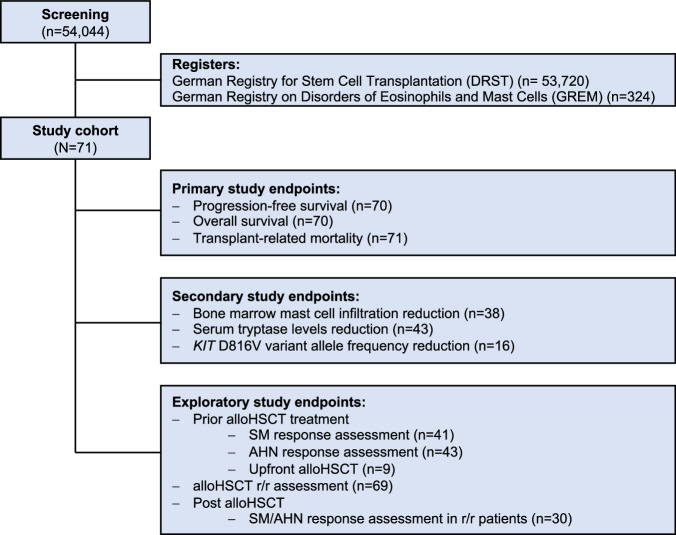
Study profile. Patients were selected from the ´German Stem Cell Transplantation Registry‘ (DRST) or from the ´German Registry on Eosinophils and Mast Cells‘ (GREM).

The study cohort consisted of 71 patients who had undergone alloHCT for AdvSM in 20 German transplant centers between 1999 and 2021 accumulating into a 372 and 211 patient-years overall follow-up period since time of diagnosis and start of alloHCT, respectively. Patient data were collected from the DRST (*n* = 65) with additional data from the GREM (*n* = 6). Nine patients (8%) were previously reported by Ustun et al. [26]. Data collection from the registries was performed by query of ´SM + alloHCT‘ in June 2022. Eligibility criteria included: (i) diagnosis of ASM, SM-AHN, SM with an acute myeloid leukemia (AML) as subtype of SM-AHN or MCL ± AHN according to the WHO-HAEM5 criteria, (ii) alloHCT and (iii) enrollment in either the DRST and/or GREM registry (Fig. 1). Following approval from the respective institutional research boards, we collected transplant data from MED-A forms, and contacted the participating centers to obtain additional information on patients’ diagnosis, specific parameters of SM, e.g. bone marrow (BM) mast cell (MC) infiltration, serum tryptase level, *KIT* D816V variant allele frequency (VAF), treatment prior to and after alloHCT, and outcome data (response, PFS and OS). Individual information was collected pseudo-anonymously using a data collection form, and transferred to the data collection centers (University Hospitals Aachen and Mannheim). The mastocytosis reference and transplant centers Aachen and Mannheim carried out a post-hoc manual review of the data for identification of inconsistencies and outliers, which were confirmed or revised in collaboration with the reporting center. Classification of conditioning regimes in myeloablative (MAC) or reduced intensity conditioning (RIC) regimes followed proposed definitions based on the duration of cytopenia and need of stem cell support [30]. Informed patient consent was given by all patients via the two registries. The study design adhered to the principles outlined in the Declaration of Helsinki and was approved by the ethics committee of the Medical Faculties Aachen and Mannheim (Germany).

### Study endpoints

Primary study endpoints included PFS and OS defined as time from date of alloHCT to date of progression of the SM and/or the AHN component, death or date of last follow-up (if progression-free) and time from date of alloHCT to death or date of last follow-up (if alive), relapse-related mortality (RRM) and NRM. Secondary endpoints included changes in the BM MC infiltration, serum tryptase levels, and *KIT* D816V VAF. Exploratory endpoints considered type, number of lines and principal investigator-assessed response to treatment prior to and after alloHCT, assessed qualitatively (response vs. non-response), and reviewed by the data collection center. Due to the limitations of retrospective, multicentric data collection, standardized response criteria such as the modified Valent criteria, International Working Group-Myeloproliferative Neoplasms Research and Treatment (IWG-MRT) ECNM criteria or Pure Pathological Response criteria could not be utilized [20, 31–33].

### Statistical analysis

Statistical analyses performed on clinical, laboratory and molecular parameters were obtained at the time of diagnosis/first referral to the servicing center and throughout the disease course. Continuous variables were analyzed for statistical differences using the Student’s *t*-test. If the values were not normally distributed, a Wilcoxon rank-sum test was employed. For categorical variables, Fisher’s exact test was used. For the estimation of hazard ratios (HRs) and multivariable analyses, the cox proportional hazard regression model was used. Only variables that demonstrated statistical significance in univariate analyses were incorporated into the multivariable model. Survival probabilities (PFS and OS) were calculated by the Kaplan-Meier Method and compared by the log-rank test. The proportional hazards assumption was tested by the correlation of scaled Schoenfeld residuals with time. The cumulative incidence function was used to account for the presence of competing risks (RRM vs. NRM). In this hypothesis-driven, retrospective data set, analyses were not specifically powered for direct comparisons. In general, a test result with P less than .05 was considered as statistically significant. Statistical analyses were performed using R version 4.3.1 (R Foundation for Statistical Computing, Vienna, Austria), SPSS version 29.0.1 (IBM Corporation, Armonk, NY) and GraphPad Prism version 10.1.1 software (GraphPad Software Inc., San Diego, CA, USA).

## Results

### Patient characteristics

At time of alloHCT, the median patient age was 59 years (range 21-84), with the majority of patients (49/71, 69%) being male. The median time between diagnosis of AdvSM and alloHCT was 1.2 (range 0.1-16.7) years. The Karnofsky performance score at time of alloHCT was ≥90% in 37/63 (59%) patients. The most common subtype close to alloHCT was ASM/SM-AHN in 30/71 (42%; ASM, *n* = 4, 6%; SM-AHN, *n* = 26, 37%), followed by SM-AML (Table S1) in 28/71 (39%, 18/28 [64%] with secondary AML) and MCL ± AHN (Table S2) in 13/71 (18%, 3/13 [23%] with secondary MCL). Diagnoses of (secondary) AML and MCL were mutually exclusive.

The detailed clinical characteristics of the various cohorts are reported in Table 1. Differences included significantly higher levels of BM MC infiltration (median 80% [range 30–100], *P* < 0.001) and numerically higher serum tryptase levels (median 429 µg/L [range 61–2660], *P* = 0.076) in MCL ± AHN while the relative frequency of the *KIT* D816V mutation (7/13, 54%, *P* < 0.001; Table S3) and of additional somatic mutations in *S/A/R* (2/10, 20%, *P* = 0.023) was significantly lower. The presence of an aberrant karyotype [23/62, 37%, including 2 patients with a t(8;21)(q22;q22)] was clustered in SM-AML (15/27, 56%, *P* = 0.008).

**Table 1:** Patient characteristics in different AdvSM subtypes.

	All	ASM/SM-AHN	SM-AML	MCL ± AHN
Number of patients, *n* (%)	71 (100)	30 (42)	28 (39)	13 (18)
Age in years at Dx; median (range)	58 (21–84)	57 (27–67)	59 (21–84)	51 (27–74)
Age in years at alloHCT; median (range)	59 (21–84)	59 (31–68)	60 (22–84)	55 (28–75)
Male, *n* (%)	49/71 (69)	21/30 (70)	19/28 (68)	9/13 (68)
Disease characteristics before alloHCT				
Karnofsky performance score ≥90%, *n* (%)	37/63 (59)	18/27 (67)	12/25 (48)	7/11 (64)
Serum tryptase ≥125 µg/L, *n* (%)	18/44 (41)	6/16 (38)	6/16 (38)	6/12 (50)
Bone marrow mast cell infiltration ≥20%, *n* (%)	27/40 (68)	10/17 (59)	7/15 (47)	11/12 (92)
* KIT* D816V positivity, *n* (%)	52/62 (84)	22/24 (92)	23/25 (92)	7/13 (54)
* SRSF2/ASXL1/RUNX1* positivity, *n* (%)	25/48 (52)	12/19 (63)	11/19 (58)	2/10 (20)
Cytogenetics, aberrant, *n* (%)	23/62 (37)	5/23 (22)	15/27 (56)	3/12 (25)
Cytogenetics, complex aberrant, *n* (%)	9/62 (15)	2/23 (9)	4/27 (15)	3/12 (25)
Disease course before alloHCT				
Lines of therapies; median (range)	2 (0–5)	1 (0–4)	2 (0–5)	3.5 (1–5)
Involvement of tyrosine kinase inhibitors, *n* (%)	35/71 (49)	14/30 (47)	9/28 (32)	12/13 (92)
Involvement of midostaurin/avapritinib, *n* (%)	27/71 (38)	10/30 (33)	5/27 (19)	11/13 (85)
Years to alloHCT since AdvSM Dx; median (range)	1.2 (0.1–16.7)	1.5 (0.3.16.7)	1.0 (0.0–12.7)	1.2 (0.2–12.7)
Years to alloHCT since AHN/AML Dx; median (range)	1.1 (0.1–16.7)	1.6 (0.2-16.7)	0.3 (0.1–3.1)	1.0 (0.2–12.7)

*AHN* associated hematologic neoplasm; *alloHCT* allogeneic hematopoietic stem-cell transplantation, *ASM* aggressive systemic mastocytosis, *Dx* diagnosis, *n* number, *SM-AHN* systemic mastocytosis with an associated hematologic neoplasm, *SM-AML* systemic mastocytosis with an acute myeloid leukemia, *MCL* *±* *AHN* mast cell leukemia with/without an associated hematologic neoplasm.

An upfront alloHCT (median time between AdvSM diagnosis and alloHCT 1.4 years, range 0.1–3.7) without prior SM- or AHN-directed treatment was performed in 9/71 (13%) patients (Table S4). All other patients received a median of 2 (range 1–5) treatment lines prior to alloHCT. Tyrosine kinase inhibitors (TKI) including midostaurin and avapritinib were less frequently used in SM-AML (9/28, 32%) than in ASM/SM-AHN (14/30, 47%, *P* = 0.259) or MCL ± AHN (12/13, 92%, *P* = 0.005) (Table 1).

### Transplant characteristics

Peripheral blood was used as graft source in 67/71 (94%), BM in 4/71 (6%) patients. Detailed donor information was available in 62/71 (87%) patients: 17 (27%) patients received a graft from an HLA-matched (10/10) related donor, 1 (2%) an HLA-mismatched related graft, 25 (40%) an HLA-matched (10/10) unrelated, 12 (19%) an HLA-mismatched unrelated, and 7 (11%) a related haploidentical graft. Overall, 24 different conditioning regimens were utilized with myeloablative conditioning accounting for 43/69 (62%) and reduced-intensity conditioning for 26/69 (38%) patients (Table S5). The most frequently administered conditioning regime consisted of fludarabine and treosulfan or fludarabine and TBI (each 9/68, 13%), followed by busulfan and cyclophosphamide (7/68, 10%).

Total body irradiation as part of the conditioning regimen, varying between 2 and 12 Gy ( ≥ 8 Gy, 11/17, 65%), was included in 17/69 (25%) patients **(**Table S6**)**. The median time to neutrophil engraftment ( ≥ 0.5/nL) was 18 days (range 1–57). Primary graft failure was reported in two patients. Data about chimerism were available in 43/71 (61%) patients. Complete donor chimerism was achieved in 36/43 (84%) patients. Acute (grade I to IV) and chronic (mild to severe) GvHD occurred in 34/67 (51%) and 16/52 (31%) patients, respectively (Table 2).

**Table 2:** Transplant characteristics in different AdvSM subtypes.

	All	ASM/SM-AHN	SM-AML	MCL ± AHN
Number of patients, *n* (%)	71 (100)	30 (42)	28 (39)	13 (19)
*Transplant*				
Graft source				
PBSC, *n* (%)	67/71 (94)	29/30 (97)	26/28 (93)	10/13 (92)
BM, *n* (%)	4/71 (6)	1/30 (3)	2/28 (7)	1/13 (8)
Conditioning				
Myeloablative, *n* (%)	43/69 (62)	16/28 (57)	16/28 (57)	11/13 (85)
Reduced intensity, *n* (%)	26/69 (38)	12/28 (43)	12/28 (43)	2/13 (15)
Total body irradiation, *n* (%)	17/70 (24)	5/29 (17)	6/28 (21)	6/28 (21)
≥8 Gy, *n* (%)	11/17 (65)	3/29 (10)	4/6 (67)	2/28 (7)
Donor				
MUD, *n* (%)	25/62 (43)	12/26 (46)	11/24 (46)	2/12 (17)
MMUD, *n* (%)	12/62 (19)	2/26 (8)	7/24 (29)	3/12 (25)
MRD, *n* (%)	17/62 (27)	9/26 (35)	3/24 (13)	5/12 (42)
MMRD, *n* (%)	1/62 (2)	0/26 (0)	1/24 (4)	0/12 (0)
Haploidentical, *n* (%)	7/62 (11)	3/26 (12)	2/24 (8)	2/12 (17)
Recipient-donor sex mismatched, *n* (%)	27/66 (41)	13/28 (46)	11/26 (42)	3/12 (25)
Donor age in years at alloHCT; median (range)	33 (17–63)	33 (19–62)	35 (22–63)	48 (17–59)
*Graft versus host disease*				
Acute graft versus host disease				
I, *n* (%)	12/67 (18)	7/28 (25)	2/27 (7)	3/13 (23)
II, *n* (%)	10/67 (15)	5/28 (18)	2/27 (7)	3/13 (23)
III, *n* (%)	8/67 (12)	3/28 (11)	3/27 (10)	1/13 (8)
IV, *n* (%)	4/67 (6)	1/28 (4)	2/27 (7)	2/13 (15)
Chronic graft versus host disease				
None, *n* (%)	36/52 (69)	13/22 (59)	12/19 (63)	11/11 (100)
Limited, *n* (%)	13/52 (25)	8/22 (36)	5/19 (26)	0/11 (0)
Extensive, *n* (%)	3/52 (6)	1/22 (5)	2/19 (11)	0/11 (0)
Prophylaxis				
Methorexate, *n* (%)	24/68 (35)	12/28 (43)	7/27 (27)	5/13 (36)
Cyclosporine, *n* (%)	58/68 (85)	24/28 (86)	25/27 (93)	9/13 (69)
Tacrolimus, *n* (%)	10/68 (15)	5/28 (18)	2/27 (7)	3/13 (23)
Mycofenolat mofetil, *n* (%)		16/28 (57)	15/27 (56)	6/13 (46)

*AHN* associated hematologic neoplasm, *alloHCT* allogeneic hematopoietic stem-cell transplantation, *AML* acute myeloid leukemia, *ASM* aggressive systemic mastocytosis, *BM* bone marrow, *MCL* *±* *AHN* mast cell leukemia with/without an associated hematologic neoplasm, *MMRD* HLA-mismatched related donor, *MMUD* HLA-mismatched unrelated donors, *MUD* HLA-matched unrelated donor, *MRD* HLA-matched related donor, *PBSC* peripheral blood stem cell, *SM-AHN* systemic mastocytosis with an associated hematologic neoplasm.

### Transplant outcome

**Fig. 2:**
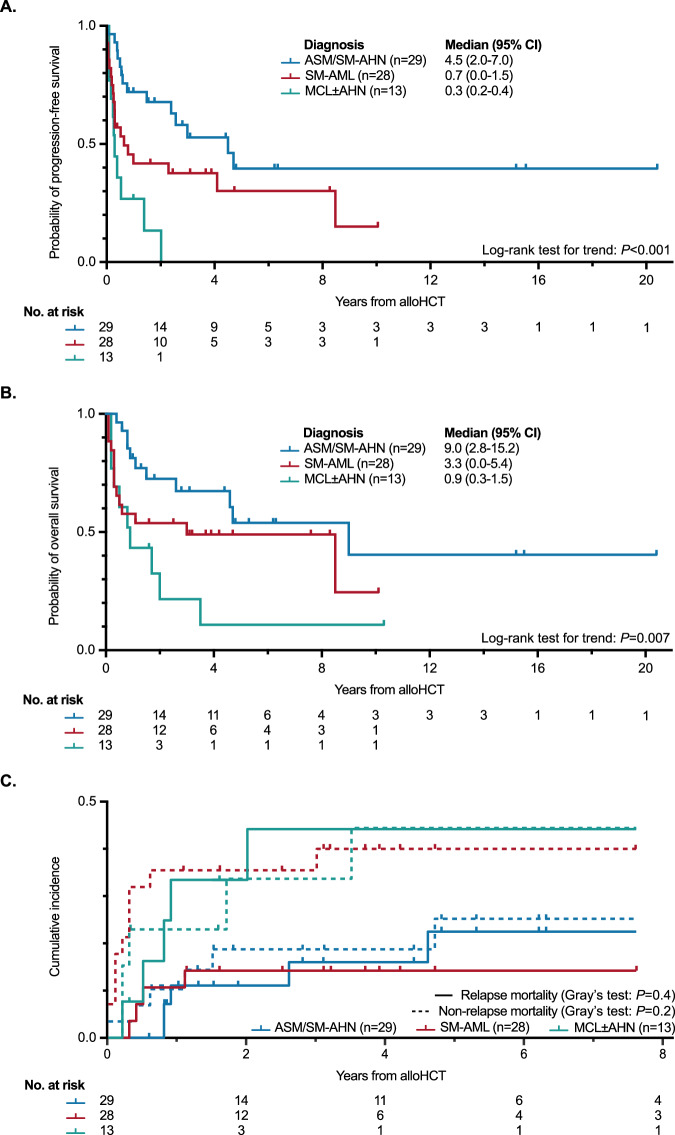
Survival outcomes. **A** Kaplan–Meier estimates of progression-free survival (PFS) and **B** overall survival (OS) depending on their most advanced disease subtype prior allogeneic stem cell transplantation (alloHCT). **C** Cumulative incidence function of relapse-related mortality and non-relapse mortality. ASM aggressive systemic mastocytosis, MCL±AHN mast cell leukemia with/without an associated hematologic neoplasm, SM-AHN systemic mastocytosis with an associated hematologic neoplasm, SM-AML systemic mastocytosis with an acute myeloid leukemia.

**Fig. 3:**
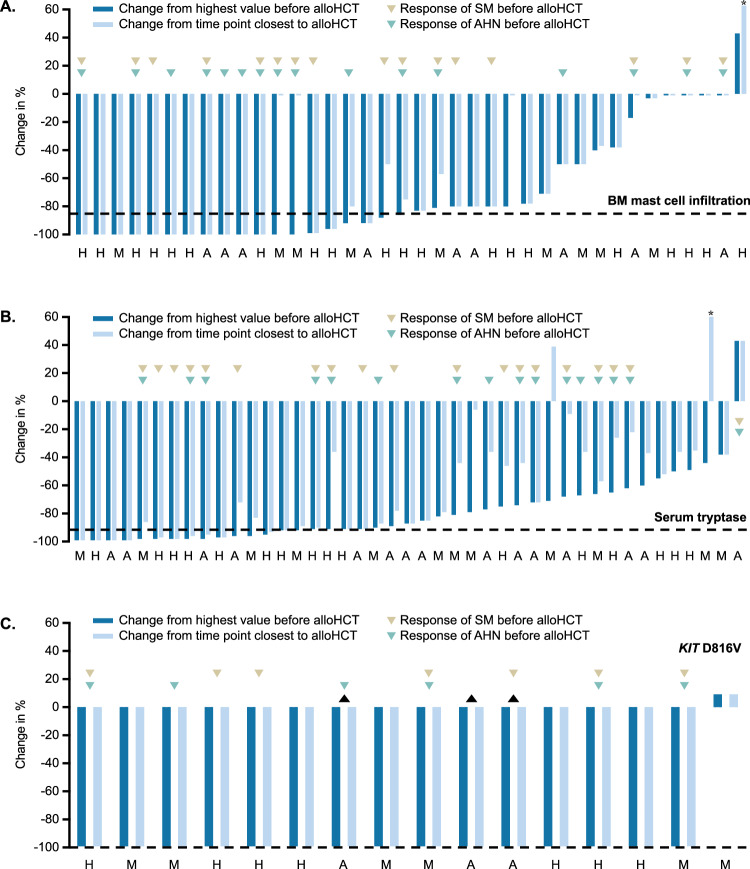
Response assessment. Waterfall plots of **A** bone marrow (BM) mast cell infiltration, **B** serum tryptase and **C** KIT D816V variant allele frequency in all available patients (relative changes prior to post alloHCT). A systemic mastocytosis with an acute myeloid leukemia, alloHCT allogeneic stem cell transplantation, AHN associated hematologic neoplasm, H systemic mastocytosis with/without an associated hematologic neoplasm (except AML), M mast cell leukemia with/without an associated hematologic neoplasm, SM systemic mastocytosis.

For the entire cohort, the median time of follow-up was 1.4 years (range 0–20.4). PFS and OS for all patients were 52% (standard deviation [SD] ± 6.1%) and 62% (SD ± 5.9%) at 1 year, and 39% (SD ± 6.3%) and 50% (SD ± 6.2%) at 3 years, respectively. Primary diagnosis of ASM/SM-AHN vs. SM-AML vs. MCL ± AHN delineated a three-tier risk stratification (median PFS, 4.5 vs. 0.7 vs. 0.3 years, *P* < 0.001, Fig. 2A; median OS, 9.0 vs. 3.3 vs. 0.9 years, *P* = 0.007, Fig. 2B). Median relative decreases in BM MC infiltration (−79% [range −100 to +233]), serum tryptase levels (−78% [range −99 to +385]) and *KIT* D816V VAF (-100% [range −100 to +10]) prior to post-alloHCT were not statistically different between the three subgroups (Fig. 3).

Taking into account competing events at years 1, 3, and 5, the RRM and the NRM rate was 15%/23%, 20%/30% and 23%/35%, respectively. Reasons for NRM included infections, GvHD, organ toxicity (cardiotoxicity) and combinations/not further specified in 8/22 (36%), 1/22 (5%), 1/22 (5%) and 11/22 (50%) patients, respectively. No statistical differences were observed in RRM (*P* = 0.400) and NRM (*P* = 0.200) based on the subtype of AdvSM (Fig. 2C, Table S7).

### Predictors of transplant outcome

Univariate analysis revealed that PFS after alloHCT was adversely impacted by diagnosis of MCL ± AHN (HR 3.5 [95% CI 1.7–7.5], *P* < 0.001), absence of a *KIT* D816V mutation (HR 2.5 [95% CI 1.2–5.4], *P* = 0.021), presence of a complex karyotype (HR 3.0 [95% CI 1.4–6.6], *P* = 0.006) and absence of the use of TKI prior to alloHCT (HR 0.5 [95% CI 0.2-0.9], *P* = 0.014). Multivariable analysis revealed the absence of the use of TKI prior to alloHCT (HR 2.8 [95% CI 1.3–5.9], *P* = 0.007) and a complex karyotype (HR 3.3 [95% CI 1.5–7.2], *P* = 0.004) as independent predictors for PFS.

**Fig. 4:**
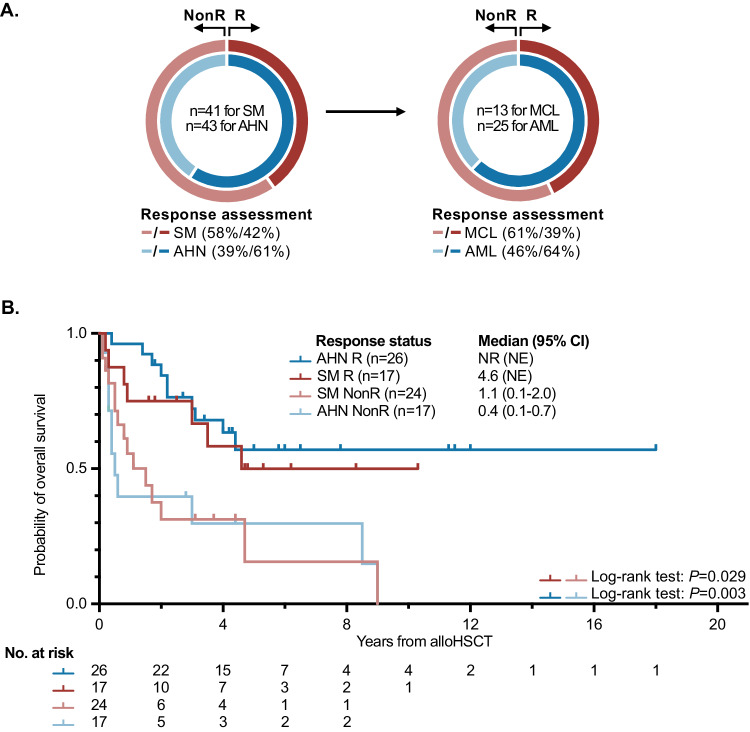
Survival outcomes based on response assessment. **A** Response status (investigator-assessed) prior to allogeneic stem cell transplantation (alloHCT) regarding the systemic mastocytosis (SM) and associated hematologic neoplasm (AHN) compartment. **B** Kaplan–Meier estimates of overall survival (OS) depending on response status prior to alloHCT. AML acute myeloid leukemia, MCL mast cell leukemia, NonR nonresponse, R response.

An adverse impact on OS was unveiled by univariate analyses upon lack of response on SM (response in 17/41, 41%; median OS 4.6 vs. 1.1 years; HR 2.5 [95% CI 1.1–6.0], *P* = 0.035) or AHN (response in 26/43, 60%, median OS not reached vs. 0.4 years; HR 0.302 [95% CI 0.132–0.687], *P* = 0.004) prior to alloHCT (Fig. 4). Transplant-associated OS was further adversely associated by the absence of a *KIT* D816V mutation (HR 2.8 [95% CI 1.2–6.5], *P* = 0.016) and a complex karyotype (HR 4.2 [95% CI 1.8–10.0], *P* = 0.001). Multivariable analysis revealed the lack of AHN response prior to alloHCT (HR 4.082 [95% CI 1.7–9.8], *P* = 0.002) and a complex karyotype (HR 8.0 [95% CI 2.3–27.1], *P* = 0.016) as independent adverse prognostic factors for OS.

HLA-matching (complete vs. incomplete), type of conditioning (myeloablative vs. dose-reduced intensity), the use of total body irradiation ( ≥ 8 Gy yes vs. no), transplantation at a center with an above-average number of alloHCT in AdvSM patients (center performed and reported ≥7 alloHCTs for AdvSM yes vs. no) or a transplantation in the year 2010 or later (Table S8) were not associated with differences in PFS or OS (Table 3).

**Table 3:** Factors associated with impacted/improved overall survival and progression-free survival.

		Overall survival			Progression-free survival	
Factors	*n*	*HR (95% CI)*	*P*	*n*	*HR (95% CI)*	*P*
**Demographics**						
Age (years) < 60 vs. ≥ 60	70	1.1 (0.6–2.1)	0.768	70	1.2 (0.7–2.1)	0.589
Sex male vs. female	70	1.3 (0.7–2.7)	0.434	70	1.2 (0.6–2.3)	0.623
Karnofsky index ≤90% vs. >90%	70	1.4 (0.7–2.8)	0.363	70	1.3 (0.7–2.4)	0.437
**Diagnosis before alloHCT**						
Diagnosis of MCL ± AHN yes vs. no	70	2.0 (0.9–4.4)	0.088	70	**3.5 (1.7–7.5)**	**<0.001**
Diagnosis of AML yes vs. no	70	1.4 (0.7–2.7)	0.309	70	1.3 (0.7–2.3)	0.401
**Cytogenetics**						
Aberran yes vs. no	60	1.6 (0.8–3.3)	0.188	60	1.6 (0.8–3.1)	0.156
Complex aberrant yes vs. no	**60**	**4.2 (1.8–10.0)**	**0.001**	**60**	**3.0 (1.4–6.6)**	**0.006**
**Mutations**						
Absence of KIT D816V yes vs. no (or unknown)	**61**	**2.8 (1.2–6.5)**	**0.016**	**61**	**2.5 (1.2–5.4)**	**0.021**
Presence of S/A/R yes vs. no	47	0.8 (0.4–1.7)	0.599	56	0.6 (0.3–1.2)	0.146
≥3 additional somatic mutations yes vs. no	56	1.3 (0.6–2.8)	0.432	56	1.0 (0.5–2.1)	0.904
**Treatment before alloHCT**						
With tyrosine kinase inhibitors yes vs. no	70	0.6 (0.3–1.1)	0.091	**70**	**0.5 (0.2–0.9)**	**0.014**
**Response status before alloHCT**^**a**^						
For SM yes vs. no	**41**	**0.4 (0.2–0.9)**	**0.035**	41	0.6 (0.3–1.2)	0.143
For AHN including AML yes vs. no	**43**	**0.3 (0.1–0.7)**	**0.004**	43	0.5 (0.2–1.0)	0.067
**Time to alloHCT**						
From SM (years) ≥1 vs. <1	70	1.0 (0.5–1.9)	0.946	70	0.8 (0.4–1.4)	0.377
From AHN (years) ≥1 vs. <1	70	1.5 (0.7–3.0)	0.297	70	1.4 (0.7–2.6)	0.332
**Biomarkers at alloHCT**						
Tryptase (µg/L) ≥100 vs. <100	50	0.9 (0.4–1.9)	0.748	50	1.0 (0.5–2.1)	0.928
Mast cell infiltration (%) ≥25 vs. <25	53	1.3 (0.6–2.7)	0.468	53	1.7 (9.9–3.4)	0.123
**Transplantation**						
Conditioning Reduced intensity vs. myeloablative	69	0.9 (0.5–1.8)	0.871	69	0.9 (0.5–1.7)	0.802
Total body irradiation ≥ 8 Gy yes vs. no	17	0.9 (0.2–3.1)	0.807	17	0.7 (0.2–2.1)	0.480
HLA match 10/10 vs. others	62	1.2 (0.6–2.5)	0.571	62	1.2 (0.6–2.4)	0.527
alloHCT before 2009 yes vs. no	70	1.1 (0.4–3.1)	0.799	70	1.3 (0.5–3.5)	0.545
alloHCT at a center with above-average numbers of alloHCT in AdvSM ^b^ yes vs. no	70	1.0 (0.5–2.0)	0.964	70	1.1 (0.6–2.0)	0.802
Full donor chimerism (≥95% donor signal within 30 days) yes vs. no	43	0.5 (0.1–1.9)	0.136	43	0.2 (0.1–1.6)	0.051

*alloHCT* allogeneic stem cell transplantation, *AHN* associated hematologic neoplasm, *AML* acute myeloid leukemia, *MCL* *±* *AHN* mast cell leukemia with/without an associated hematologic neoplasm, *S/A/R*, *SRSF2/ASXL1/RUNX1*; SM systemic mastocytosis.

^a^*n* = 9 patients with upfront alloHCT without any induction therapy were excluded from this analysis.

^b^Center performed and reported ≥7 alloHCT.

Significant results are highlighted in bold.

### Posttransplant treatment

During or after alloHCT, 30/71 (42%) patients showed either refractory (9/30, 30%) or relapsed (21/30, 70%; r/r) disease which was highest in MCL ± AHN (11/13, 85%) vs. ASM/SM-AHN (9/30, 30%) and SM-AML (10/28, 36%; *P* = 0.002). Primary refractory disease was predominantly observed in MCL ± AHN (8/9, 89%) vs. SM-AML (1/9, 11%) and ASM/SM-AHN, 0/9, 0%; (*P* < 0.001). The median time to relapse was 0.6 years (range 0.1–4.5) which was more frequent in ASM/SM-AHN (9/21, 43%) and SM-AML (9/21, 43%) vs. MCL ± AHN (3/21, 14%). Considering the two disease components, r/r SM was predominantly observed in MCL ± AHN (10/11, 91%), r/r AHN was higher in SM-AML (6/10, 60%) and ASM/SM-AHN (5/9, 56%).

Overall, 52 treatment lines have been applied in the r/r setting post-alloHCT with a median number of 2 (range 0–4) lines per individual patient. At least one treatment line was applied in 24/30 (80%) patients. Midostaurin/avapritinib, cladribine or other cytoreductive therapies were used in 9/30 (30%), 3/30 (10%) and 14/30 (47%) patients, respectively (Table 4). Donor lymphocyte infusions were applied in 18/30 (60%) patients (ASM/SM-AHN, 7/9, 78%; SM-AML, 7/10, 70%, MCL ± AHN, 4/11, 36%) while a second alloHCT was performed in 2/30 (7%) patients (both SM-AML). A response to treatment regimens after alloHCT was achieved in 13/30 (43%) patients (ASM/SM-AHN, 3/9, 33%, SM-AML, 5/10, 50%; MCL ± AHN, 5/11, 45%) and was highest in 9 patients receiving midostaurin and/or avapritinib (7/9, 78%).

**Table 4:** Post-alloHCT treatment in patients with refractory/relapse disease.

	All		ASM/SM-AHN		SM-AML		MCL ± AHN	
Number of patients, *n* (%)	30/71 (42)		9/30 (30)		10/28 (36)		11/13 (85)	
Refractory disease, *n* (%)	9/30 (30)		0/9 (0)		1/10 (10)		8/11 (73)	
SM, *n* (%)	8/9 (89)		–		0/1 (0)		8/8 (100)	
AHN, *n* (%)	0/9 (0)		–		0/1 (0)		0/8 (0)	
SM + AHN, *n (%)*	1/9 (11)				1/1 (100)		0/8 (0)	
Relapse disease, *n* (%)	21/30 (70)		9/9 (100)		9/10 (90)		3/11 (27)	
SM, *n* (%)	8/21 (38)		4/9 (44)		2/9 (22)		2/3 (67)	
AHN, *n* (%)	12/21 (57)		5/9 (56)		6/9 (67)		1/3 (33)	
SM + AHN, *n* (%)	1/21 (5)		0/9 (0)		1/10 (10)		0/3 (0)	
Years to relapse, median (range)	0.6 (0.1-4.5)		0.6 (0.3-4.5)		0.4 (0.1-4.1)		1 (0.4-1.4)	
**Post-alloHCT treatment**	***n*** **(%)**	**Response**, ***n*** **(%)**	***n*** **(%)**	**Response**, ***n*** **(%)**	***n*** **(%)**	**Response**, ***n*** **(%)**	***n*** **(%)**	**Response**, ***n*** **(%)**
Lines of therapy, median (range)	2 (0–4)	–	1 (1–4)	–	2 (0–4)	–	2 (0–4)	–
Tyrosine kinase inhibitors								
Midostaurin	8/30 (27)	4/8 (50)	1/9 (11)	1/1 (100)	2/10 (20)	2/2 (100)	5/11 (45)	1/5 (20)
Avapritinib	4/30 (13)	3/4 (75)	0/9 (0)	–	0/10 (0)	–	4/11 (36)	3/4 (75)
Other TKI^a^	3/30 (10)	0/3 (0)	1/9 (11)	0/1 (0)	1/10 (10)	0/1 (0)	1/11 (9)	0/1 (0)
Cytoreductive therapy								
Cladribine	3/30 (10)	0/3 (0)	1/9 (11)	0/1 (0)	1/10 (10)	0/1 (0)	1/11 (9)	0/1 (0)
Others^b^	14/30 (47)	5/14 (36)	5/9 (56)	2/5 (40)	5/10 (50)	3/5 (60)	4/11 (36)	0/4 (0)
Donor lymphocyte infusion	18/30 (60)	5/18 (28)	7/9 (78)	1/7 (14)	7/10 (70)	3/7 (43)	4/11 (36)	1/4 (25)
Second alloHCT	2/30 (7)	2/2 (100)	0/9 (0)	–	2/10 (20)	2/2 (100)	0/11	–
**Response to post-alloHCT treatment**^**c**^								
Response, *n* (%)	13/30 (43)		3/9 (33)		5/10 (50)		5/11 (45)	
No response or progression, *n* (%)	10/30 (33)		3/9 (33)		3/10 (30)		4/11 (36)	

*alloHCT* allogeneic stem cell transplantation, *ASM* aggressive systemic mastocytosis, *DLI* donor lymphocyte infusion, *MCL* *±* *AHN* mast cell leukemia with/without an associated hematologic neoplasm, *r/r* relapsed/refractory, *SM-AHN* systemic mastocytosis with an associated hematologic neoplasm, *TKI* tyrosine kinase inhibitor.

^a^dasatinib, imatinib, nilotinib.

^b^azacitidine, bortezomib, decitabine, hydroxyurea, induction chemotherapy, nivolumab, venetoclax.

^c^Overall response independent of response in either the SM and/or the AHN compartment.

## Discussion

Despite the availability and efficacy of KIT targeted therapies, alloHCT remains the only curative treatment option for patients with AdvSM. Our data show that primary disease phenotype, karyotype and endpoints assessed during follow-up such as level of response as well as primary or secondary resistance are critical for outcome of subsequently performed alloHCT. So far, a retrospective multicenter analysis from 2014 including 57 patients (transplanted between 1990 and 2013) represents the only available data collection demonstrating a potential curative benefit of alloHCT in AdvSM [26]. Applying these reported findings into current clinical practice remains however challenging given the increasing complex molecular landscape of AdvSM, the efficacy of KIT inhibitors such as midostaurin and avapritinib, and the continuous developments of transplant procedures, GvHD prophylaxis and supportive care strategies [18–20, 22–25, 34]. A prospective clinical trial in order to assess the best alloHCT strategy will most likely not be performed given the rarity and heterogeneity of AdvSM. Ongoing clinical trials of new KIT inhibitors such as bezuclastinib (NCT04996875) or elenestinib (NCT05609942) pose additional potential challenges on the evaluation of optimal alloHCT integration into the overall treatment concepts. However, in order to address some of the actual questions in regard to alloHCT in AdvSM patients we performed an updated retrospective multicenter study on 71 AdvSM patients who underwent alloHCT in Germany between 1999 and 2021 with 89% of patients transplanted after 2010.

As expected, substantial disparities were noted between the various AdvSM subtypes with a median OS of approximately 1 and 3 years observed in MCL ± AHN and SM-AML, respectively. The most inherent characteristics contributing to this unfavorable prognosis include the absence of *KIT* D816V in approximately 50% of MCL patients and the presence of an aberrant karyotype in 56% or a complex karyotype in 15% of patients with SM-AML. Surprisingly, PFS/OS was not adversely affected by established high-risk mutations (HRM) such as *SRSF2, ASXL* or *RUNX1* [8, 10, 11, 18, 34, 35]. In ASM/SM-AHN patients, a median OS of nine years was seen after exclusion of MCL and AML. These data clearly indicates that alloHCT can overcome poor prognosis conferred by HRM in *KIT* D816V positive AdvSM at least if MCL or AML are absent.

Similar to AML [36–38] or ALL [39], response of the SM and/or the AHN compartment to treatment prior alloHCT significantly prolonged transplant-associated OS. Our data also show that for patients with resistant or progressive disease before alloHCT, this procedure might not be a reasonable rescue option as the graft-versus-AdvSM effect does not seem to be sufficiently effective in patients with high disease burden, a phenomenon known from other myeloid neoplasms [40–42]. To optimize outcome in AdvSM, transplant eligible patients should therefore be transplanted at time of best response to pre-allo treatment; hence, close interdisciplinary cooperation between mastocytosis and transplant centers is warranted to define these time points. The use of tyrosine kinase inhibitors such as midostaurin or avapritinib prior to alloHCT was significantly associated with improved PFS. The use of 24 different conditioning regimens in 71 AdvSM patients demonstrates the lack of a standardized approach for AdvSM patients despite consensus opinions [43, 44]. This inconsistency becomes even more impressive when we consider that all transplantations have been conducted within one country.

Even after adjusting for pre-alloHCT treatment responses (data not shown), updated analyses could not confirm the previously reported superiority of myeloablative conditioning over reduced intensity regimens in terms of PFS [26]. This again highlights the fact, that other parameters such as pre-alloHCT response seem to be more important than conditioning intensity. Therefore, future strategies for older or more fragile patients should prioritize achieving an optimal treatment response before considering alloHCT to employ reduced-intensity conditioning regimens successfully.

Posttransplant treatment was exclusively used in patients with r/r disease status. As expected, the relative frequency of r/r patients with 85% was highest in patients with MCL ± AHN with most of these patients (62%) already suffering from refractory disease prior to alloHCT. As pointed out before, the use of myeloablative conditioning was insufficient to overcome this mast cell resistance. While in approximately 50% of patients with r/r MCL ± AHN, a partial response was achieved on midostaurin (*n* = 1), DLI (*n* = 1) or avapritinib (*n* = 3), future strategies might focus on maintenance therapy or measurable residual disease-based pre-emptive approaches using effective TKIs, e.g. similar to strategies used in AML patients with FLT3 mutation [45].

A major limitation of this analysis is insufficient information on post-transplant quantification of residual disease including BM MC infiltration (24/71, 34%), serum tryptase levels (33/71, 47%) and *KIT* D816V VAF (48/71, 68%) not being available in a significant number of patients. This lack of data - even as chimerism analyses on BM aspirates were regularly performed - was particular predominant in patients in which the AHN compartment was the primary driver for the alloHCT concept, e.g. SM-AML [6, 9, 18]. In consequence, residual MC disease may be inadvertently overlooked during follow-up. The lack of attention in regard to the MC component post-alloHCT could be a significant disadvantage especially given the availability of potent MC-directed treatment options such as midostaurin and avapritinib [18–20, 46–49].

Similarities and differences between the cohort reported by Ustun et al. and the current cohort are at least partly related to changes in the real-world practice of alloHCT over a 10-years period between the two reports. For example, direct comparisons across the various subtypes were not possible because advanced morphologic and genetic characterization conferred into a higher relative frequency of AHN (87% vs. 67%) in the current cohort. Moreover, the number of cases allowed the separation of SM-AML from SM-AHN because it is characterized by an inherent disease phenotype and different clinical trajectory. In the current cohort, patients were older (median age 59 vs. 46 years) and the relative frequencies of unrelated (60% vs. 31%) or haploidentical donors (11% vs. 2%) were higher while the relative frequencies on the use of MAC regimens (62% vs. 63%) and TBI (24% vs. 28%) were similar. Rates of NRM were 23% (based on cumulative incidence function) and 20% after 1 year and the subtype-independent OS was approximately 60% and 50% after 1 and 3 years in both cohorts. In this respect, it should be emphasized that the poor prognosis of MCL (with or without AHN) has not markedly improved over the last decade.

In conclusion, alloHCT can confer long-term PFS/OS in patients with AdvSM, especially when alloHCT will be performed at time of optimal response. To achieve the maximum benefit from pre- and post-allogeneic strategies and from the transplant itself, collaborative efforts between mastocytosis experts and transplant centers are essential. Adequate monitoring of residual disease could provide valuable guidance for the pre-emptive use of KIT inhibitors post-transplant. The rapidly evolving landscape of AdvSM treatment necessitates continuous adaptation in integrating alloHCT into patient care. To facilitate upcoming comprehensive data analysis, the implementation of a uniform transplantation data collection form encompassing key elements relevant to the specific characteristics and heterogeneities of AdvSM and alloHCT is of utmost importance.

### Supplementary information

Supplementary information

## Data Availability

The datasets used and/or analyzed during the current study are available from the corresponding author on reasonable request.
