# Supplementary material for: Allogeneic Hematopoietic Cell Transplantation in Advanced Systemic Mastocytosis: A retrospective analysis of the DRST and GREM registries
Source: Leukemia. 2024 Mar 6;38(4):810–21. doi: 10.1038/s41375-024-02186-x (PMC10997505; doi:10.1038/s41375-024-02186-x)
Supplement: Supplementary file 1 — Supplementary information [file 41375_2024_2186_MOESM1_ESM.docx]

**Supplementary information**

**Allogeneic Hematopoietic Cell Transplantation in Advanced Systemic Mastocytosis:**

**A retrospective analysis of the DRST and GREM registries**

Johannes Lübke, Deborah Christen, Juliana Schwaab, Anne Kaiser, Nicole Naumann,
Khalid Shoumariyeh, Madlen Jentzsch, Katja Sockel, Judith Schaffrath, Francis A. Ayuk,
Matthias Stelljes, Inken Hilgendorf, Elisa Sala, Jennifer Kaivers, Stefan Schönland,
Christoph Wittke, Bernd Hertenstein, Markus Radsak, Ulrich Kaiser, Valeska Brückl,
Nicolaus Kröger, Tim H. Brümmendorf, Wolf-Karsten Hofmann, Stefan Klein, Edgar Jost,
Andreas Reiter, Jens Panse

**Table of contents**

**Supplementary Tables**

**Table S1:** Individual characteristics of 28 patients with (secondary) acute myeloid
leukemia before or at time alloHCT…………………………………………………………….2

**Table S2:** Individual characteristics of 11 patients with diagnosis of mast cell leukemia
before or at time alloHCT…………………………………………...……………………………3

**Table S3:** Patient and transplant characteristics depended on the presence/absence of
the *KIT* D816V mutation………………………………………………………………………….4

**Table S4:** Individual characteristics of 9 patients with upfront alloHCT……….……………5

**Table S5:** Individual conditioning regime…………………………...…………………………6

**Table S6:** Total body irradiation in 17 patients.………………………………….………...….8

**Table S7:** Competing risk analysis of relapse and non-relapse mortality…………….…....9

**Table S8:** Transplant characteristics in different AdvSM subtypes across different time
periods …………………………………………………………………………………..….…...10

| **Table S1: Individual characteristics of 28 patients with (secondary) acute myeloid leukemia before or at time alloHCT** | | | | | | | | | | | | | | |
| --- | --- | --- | --- | --- | --- | --- | --- | --- | --- | --- | --- | --- | --- | --- |
| **#** | **Sex** | **Age (y) at alloHCT** | **AML at AdvSM Dx** | **Initial AHN Dx** | **ELN risk group 2022** | **Dx of MCL** | ***KIT* D816V** | **Tryptase (µg/L)** | | **BM MC infiltration (%)** | | **Additional mutations ^b^** | **Karyotype** | |
|  |  |  |  |  |  |  |  | **highest** | **at alloHCT** | **highest** | **at alloHCT** |  |  |  |
| 1 | M | 67 | No | HES/CEL | Adverse | No | Positive | - | - | 10 | 10 | *ASXL1, JAK2,* | Abnormal | (c.a.) |
| 6 | F | 56 | Yes | None ^a^ | Adverse | No | - | - | - | - | - | *ASXL1, KRAS, RUNX1* | Normal |  |
| 7 | F | 61 | Yes | - | Favorable | No | Positive | 200 | 94 | 18 | 15 | *None* | Abnormal^c^ |  |
| 9 | M | 59 | Yes | - | Intermediate | No | Positive | - | - | 20 | 20 | *KMT2A::PTD* | Abnormal |  |
| 19 | M | 58 | No | CMML | - | No | Positive | 37 | 18 | 25 | 25 | *-* | Normal |  |
| 23 | M | 65 | No | CMML | Favorable | No | Positive | 562 | 18 | - | - | *NPM1* | Abnormal |  |
| 26 | M | 65 | Yes | - | Intermediate | No | - | - | - | - | - | *IDH2* | Normal |  |
| 28 | M | 22 | Yes | - | Favorable | No | Positive | - | - | - | - | *NRAS* | Abnormal^c^ |  |
| 30 | M | 56 | No | MDS/MPNu | Intermediate | No | Positive | 30 | 1 | 50 | 1 | *None* | Abnormal |  |
| 32 | M | 74 | No | MDS | Adverse | No | Positive | - | - | - | - | *CBL, EZH2, KRAS, TET2* | Abnormal |  |
| 33 | M | 58 | No | CML | Adverse | No | Positive | 24 | 11 | 25 | 25 | *BCR::ABL, DNMT3A, SF3B1, TET2* | Abnormal |  |
| 35 | M | 61 | No | PMF | Adverse | No | Positive | 22 | 14 | 25 | 25 | *JAK2, RUNX1* | Abnormal | (c.a.) |
| 36 | M | 84 | No | CMML | Adverse | No | Positive | 200 | 200 | 65 | 65 | *ASXL1, KRAS, RUNX1, TET2, U2AF1* | Abnormal |  |
| 39 | F | 55 | Yes | - | Intermediate | No | Positive | - | - | 0 | 0 | *DNMT3A, FLT3-ITD, NPM1* | Normal |  |
| 44 | F | 45 | Yes | - | - | No | - | - | - | - | - | *-* | Normal |  |
| 46 | M | 61 | Yes | - | Favorable | No | Positive | 282 | 146 | 60 | 20 | *NPM1, SF3B1* | Normal |  |
| 47 | M | 47 | No | MDS/MPNu | Adverse | No | Positive | 82 | 82 | 10 | 10 | *ASXL1, BCOR, IDH2, KMT2A::PTD, RUNX1* | Abnormal |  |
| 48 | F | 68 | Yes | - | Favorable | No | Positive | 73 | 26 | 20 | 20 | *NPM1, DNMT3A* | Normal |  |
| 49 | M | 68 | No | MDS/MPNu | Adverse | No | Positive | 293 | 293 | 5 | 5 | *SRSF2, TET2* | Normal |  |
| 52 | M | 61 | Yes | - | Adverse | No | Positive | - | - | - | - | *SRSF2, TET2* | Abnormal^d^ |  |
| 55 | M | 58 | Yes | - | - | No | Positive | 200 | 167 | - | - | *RUNX1, SRSF2, TET2* | - |  |
| 56 | F | 53 | No | MDS/MPNu | Favorable | No | Negative | 4 | 4 | 6 | 6 | *NPM1* | Normal |  |
| 57 | F | 51 | No | HES/CEL | Intermediate | No | Negative | 38 | 38 | 15 | 15 | *None* | Normal |  |
| 60 | F | 28 | No | None ^a^ | Adverse | No | Positive | 230 | 230 | - | - | *RUNX1* | Normal |  |
| 61 | M | 65 | No | MDS/MPNu | Adverse | No | Positive | 875 | 114 | 25 | 5 | *SRSF2, TET2* | Abnormal |  |
| 62 | M | 38 | Yes | - | Adverse | No | Positive | - | - | - | - | *SETBP1* | Abnormal^d^ | (c.a.) |
| 67 | M | 71 | No | None ^a^ | Adverse | No | Positive | 40 | 14 | 5 | 3 | *DNMT3A, FLT3-TKD, NPM1, RUNX1* | Abnormal | (c.a.) |
| 70 | F | 60 | Yes | - | Favorable | No | Positive | 9 | 9 | 2 | 2 | *DMT3A, FLT3-TKD, NPM1, TET2* | Normal |  |
| Abbreviations: alloHCT, allogeneic stem cell transplantation; AdvSM, advanced systemic mastocytosis; AML, acute myeloid leukemia; AHN, associated hematologic neoplasm; BM, bone marrow; c.a., complex aberrant; CMML, chronic myelomonocytic leukemia; Dx, diagnosis; F, female; HES/CEL, hypereosinophilic syndrome/chronic eosinophilic leukemia; M, male; MC, mast cell; MCL, mast cell leukemia; MDS/MPNu, myelodysplastic/myeloproliferative neoplasm, unclassified; y, years  ^a^ Initial diagnosis was established as ASM.  ^b^ **I**n alphabetical order.  ^c^ t(8;21)(q22;q22.1) *RUNX1-RUNX1T1.*  ^d^ Monosomy 7. | | | | | | | | | | | | | | |

| **Table S2: Individual characteristics of 11 patients with diagnosis of mast cell leukemia before or at time alloHCT** | | | | | | | | | | | | | |
| --- | --- | --- | --- | --- | --- | --- | --- | --- | --- | --- | --- | --- | --- |
| **#** | **Sex** | **Age (y) at alloHCT** | **Age (y) at MCL Dx** | **AHN Dx** | **Dx of (s)AML** | ***KIT* D816V** | **Tryptase (µg/L)** | | **BM MC infiltration (%)** | | **Additional mutations ^a^** | **Karyotype** | |
|  |  |  |  |  |  |  | **highest** | **at alloHCT** | **highest** | **at alloHCT** |  |  |  |
| 10 | F | 45 | 46 | - | No | No | 200 | 200 | 100 | 95 | *NRAS* | Abnormal | (c.a.) |
| 11 | F | 66 | 67 | ET | No | Yes | 289 | 150 | 30 | 30 | *MPL* | Normal |  |
| 12 | M | 56 | 62 | MDS | No | Yes | 156 | 123 | 60 | 25 | *None* | Normal |  |
| 13 | M | 44 | 46 | - | No | No | 660 | 76 | 80 | 80 | *KRAS* | Normal |  |
| 17 | M | 38 | 39 | - | No | No | 2660 | 1910 | - | - | *-* | Normal |  |
| 21 | M | 75 | 75 | - | No | Yes | 806 | 183 | 80 | 70 | *-* | Abnormal | (c.a.) |
| 27 | M | 43 | 56 | MDS/MPNu | No | Yes | - | - | - | - | *-* | - |  |
| 51 | M | 64 | 65 | MDS/MPNu | No | Yes | 600 | 508 | 50 | 50 | *ASXL1, RUNX1, SRSF2* | Normal |  |
| 59 | F | 48 | 49 | MDS | No | Yes | 61 | 49 | 80 | 35 | *None* | Normal |  |
| 68 | M | 63 | 65 | - | No | No | 451 | 93 | 90 | 90 | *None* | Abnormal | (c.a.) |
| 71 | M | 27 | 28 | CEL/HES | No | No | 407 | 90 | 80 | 80 | *None* | Normal |  |
| Abbreviations: alloHCT, allogeneic stem cell transplantation; AdvSM, advanced systemic mastocytosis; AML, acute myeloid leukemia; AHN, associated hematologic neoplasm; BM, bone marrow; c.a., complex aberrant; CMML, chronic myelomonocytic leukemia; Dx, diagnosis; ET, essential thrombocythemia; F, female; HES/CEL, hypereosinophilic syndrome/chronic eosinophilic leukemia; M, male; MC, mast cell; MCL, mast cell leukemia; MDS/MPNu, myelodysplastic/myeloproliferative neoplasm, unclassified; y, years  ^a^ **I**n alphabetical order. | | | | | | | | | | | | | |

| **Table S3: Patient and transplant characteristics depended on the presence/absence of the *KIT* D816V mutation** | | | | | | | | | |  |
| --- | --- | --- | --- | --- | --- | --- | --- | --- | --- | --- |
|  | | | **SM** | | |  | **AHN** | | |  |
|  | | | **Responder** | **Non-Responder** |  |  | **Responder** | **Non-Responder** |  | |
| Number of patients, *n* (%) | | | 17 (41) | 24 (59) |  |  | 26 (60) | 17 (40) |  | |
| Age in years at Dx; median (range) | | | 57 (21-74) | 53 (27-68) |  |  | 57 (21-69) | 58 (27-73) |  | |
| Age in years at alloHCT; median (range) | | | 58 (22-75) | 56 (28-68) |  |  | 60 (22-71) | 58 (28-74) |  | |
| Male, *n* (%) | | | 9/15 (60) | 17/22 (77) |  |  | 14/24 (58) | 9/16 (56) |  | |
|  |  | |  |  |  |  |  |  |  | |
| **Disease characteristics** | | |  |  |  |  |  |  |  | |
|  | Karnofsky performance score; median (range) | | 90 (80-100) | 90 (60-100) |  |  | 90 (60-100) | 90 (60-100) |  | |
|  | *KIT* D816V positive, *n* (%) | | 16/17 (94) | 16/22 (73) |  |  | 18/22 (82) | 14/15 (93) |  | |
|  | *SRSF2/ASXL1/RUNX1* positivity, *n* (%) | | 5/12 (42) | 8/19 (42) |  |  | 8/16 (50) | 9/15 (60) |  | |
|  | Abnormal cytogenetics, *n* (%) | | 6/17 (35) | 8/22 (36) |  |  | 8/24 (33) | 7/14 (50) |  | |
|  |  | Complex aberrant, *n* (%) | 2/17 (12) | 5/22 (23) |  |  | 3/23 (13) | 2/14 (14) |  | |
|  |  | |  |  |  |  |  |  |  | |
| **Before alloHCT** | | |  |  |  |  |  |  |  | |
|  | Lines of therapies; median (range) | | 3 (1-5) | 2 (0-4) |  |  | 2 (1-5) | 2 (1-5) |  | |
|  | Involvement of TKI, *n* (%) | | 13/17 (77) | 6/20 (30) |  |  | 12/26 (46) | 8/17 (47) |  | |
|  |  | Involvement of midostaurin/avapritinib, *n* (%) | 12/17 (71) | 4/20 (20) |  |  | 9/26 (35) | 6/17 (35) |  | |
|  | Years to alloHCT since AdvSM Dx; median (range) | | 1.1 (0.2-3.2) | 1.2 (0.0-15.0) |  |  | 1.2 (0.2-16.7) | 1.1 (0.2-12.7) |  | |
|  | Years to alloHCT since AHN Dx; median (range) | | 2.7 (0.3-9.7) | 1.0 (0.0-15.0) |  |  | 1.2 (0.2-16.7) | 1.1 (0.2-12.7) |  | |
|  |  | |  |  |  |  |  |  |  | |
| **Transplant** | | |  |  |  |  |  |  |  | |
|  | Graft source | |  |  |  |  |  |  |  | |
|  |  | PBSC, *n* (%) | 16/17 (94) | 24/24 (100) |  |  | 24/26 (92) | 16/17 (94) |  | |
|  |  | BM, *n* (%) | 1/17 (6) | 0/0 (0) |  |  | 2/26 (8) | 1/17 (6) |  |  |
|  | Conditioning | |  |  |  |  |  |  |  | |
|  |  | Myeloablative, *n* (%) | 10/17 (59) | 16/23 (70) |  |  | 16/26 (62) | 11/17 (65) |  | |
|  |  | Reduced intensity, *n* (%) | 7/17 (41) | 7/23 (30) |  |  | 10/26 (38) | 6/17 (35) |  |  |
|  |  | With total body irradiation, *n* (%) | 5/17 (29) | 8/24 (33) |  |  | 6/26 (23) | 5/17 (29) |  | |
|  | Donor | |  |  |  |  |  |  |  | |
|  |  | MUD, *n* (%) | 8/15 (53) | 7/21 (33) |  |  | 11/21 (52) | 4/15 (27) |  | |
|  |  | MMUD, *n* (%) | 3/15 (20) | 3/21 (14) |  |  | 6/21 (29) | 5/15 (33) |  | |
|  |  | MRD, *n* (%) | 3/15 (20) | 8/21 (38) |  |  | 2/21 (10) | 5/15 (33) |  | |
|  |  | MMRD, n (%) | 0/0 (0) | 0/0 (0) |  |  | 0/21 (0) | 0/15 (0) |  | |
|  |  | Haploidentical, *n* (%) | 1/15 (7) | 3/21 (14) |  |  | 2/21 (10) | 1/15 (7) |  | |
|  |  | Recipient-donor sex mismatched, *n* (%) | 6/15 (40) | 10/22 (46) |  |  | 7/24 (29) | 7/16 (44) |  | |
|  |  | Donor age in years at alloHCT; median (range) | 33 (19-63) | 32 (17-59) |  |  | 31 (19-59) | 28 (20-63) |  | |
|  |  | |  |  |  |  |  |  |  | |
| **Response assessment** | | |  |  |  |  |  |  |  | |
|  | Response to previous therapy ^a^ | |  |  |  |  |  |  |  | |
|  |  | SM, *n* (%) | 17/17 (100) | 0/24 (0) |  |  | 10/14 (71) | 2/11 (18) |  | |
|  |  | AHN, *n* (%) | 10/12 (83) | 4/13 (31) |  |  | 26/26 (100) | 0/17 (0) |  | |
|  | Refractory to/Relapse after alloHCT, *n* (%) | | 8/17 (47) | 13/21 (62) |  |  | 12/25 (48) | 7/15 (47) |  | |
|  |  | |  |  |  |  |  |  |  | |
| **Outcome** | | |  |  |  |  |  |  |  | |
|  | Follow-up, years since Dx; median (range) | | 4.2 (0.4-11.5) | 2.1 (0.4-15.8) |  |  | 4.1 (0.4-18.0) | 1.4 (0.4-21.2) |  | |
|  | Follow-up, years since alloHCT; median (range) | | 3.0 (0.0-10.3) | 0.8 (0.0-9.0) |  |  | 2.8 (0.1-10.3) | 0.4 (0.0-9.0) |  | |
|  | Deaths, *n* (%) | | 8/17 (0.124) | 17/24 (71) |  |  | 10/26 (39) | 14/17 (82) |  | |
|  | Median OS, years (95% CI) | | 4.6 (NE) | 1.1 (0.1-2.0) |  |  | NR (NE) | 0.4 (0.1-0.7) |  | |
| Abbreviations: AHN, associated hematologic neoplasm; alloHCT, allogeneic hematopoietic stem-cell transplantation; AML, acute myeloid leukemia; ASM, aggressive systemic mastocytosis; BM, bone marrow; CI, confidence interval; Dx, diagnosis; MCL±AHN, mast cell leukemia with/without an associated hematologic neoplasm; MMUD, HLA-mismatched unrelated donors; MUD, HLA-matched unrelated donor; MMRD, HLA-mismatched related donor; MRD, HLA-matched related donor; OS; overall survival; PB, peripheral blood; PBSC, peripheral blood stem cell; SM-AHN, systemic mastocytosis with an associated hematologic neoplasm  ^a^ Clinically assessed response according to principal investigator. ^b^ Change from highest value in disease course before alloHCT. | | | | | | | | | |  |

| **Table S4: Individual characteristics of 9 patients with upfront alloHCT** | | | | | | | | | | | | |
| --- | --- | --- | --- | --- | --- | --- | --- | --- | --- | --- | --- | --- |
| **#** | **Sex** | **Age (y) at alloHCT** | **WHO-5 Dx** | **Dx of MCL** | **Dx of AHN** | ***KIT* D816V** | **Karyotype** | **Time from Dx to alloHCT** | **Donor type** | **Post-alloHCT treatment** | **Death** | |
|  |  |  |  |  |  |  |  | in years |  |  | Time from alloHCT to last contact | Death |
| 4 | M | 65 | SM-AHN | No | No | Yes | Normal | 1.0 | MUD | DLI | 0.6 | No |
| 16 | F | 64 | SM-AHN | No | No | Yes | Abnormal | 1.4 | MUD | None | 15.2 | No |
| 25 | M | 61 | ASM | No | No | Unknown | Unknown | 1.5 | MRD | None | 6.3 | No |
| 36 | M | 84 | SM-AHN | No | No | Yes | Abnormal | 0.3 | haplo | None | 1.6 | No |
| 37 | F | 59 | SM-AHN | No | No | Yes | Normal | 2.8 | MRD | None | 1.0 | No |
| 38 | F | 61 | SM-AHN | No | No | Yes | Normal | 3.6 | haplo | DLI | 0.8 | No |
| 49 | M | 68 | SM-AHN | No | No | Yes | Normal | 3.7 | MMRD | None | 0.3 | Yes |
| 52 | M | 61 | SM-AHN | No | Yes | Yes | Abnormal | 0.0 | MUD | DLI, decitabine/venetoclax, midostaurin | 1.1 | No |
| 53 | M | 31 | SM-AHN | No | No | Unknown | Unknown | 0.6 | MUD | DLI | 20.4 | No |
| Abbreviations: alloHCT, allogeneic stem cell transplantation; AdvSM, advanced systemic mastocytosis; AML, acute myeloid leukemia; AHN, associated hematologic neoplasm; Dx, diagnosis; F, female; haplo, haploidentical; M, male; MC, mast cell; MCL, mast cell leukemia; MMRD, HLA-mismatched related donor; MUD, HLA-matched unrelated donor; MRD, HLA-matched related donor; SM, systemic mastocytosis; SM-AHN, systemic mastocytosis with an associated hematologic neoplasm; WHO, World Health Organization; y, years | | | | | | | | | | | | |

| **Table S5: Individual conditioning regime** | | | | |
| --- | --- | --- | --- | --- |
| **#** | **Dx at time of alloHCT** | **Age at alloHCT** |  | **Conditioning regime^a^** |
| 1 | SM-AHN (HES/CEL) | 67 |  | busulfan 6.4 mg/kg, cyclophosphamide 120 mg/kg |
| 2 | SM-AHN (CMML) | 61 |  | busulfan 6.4 mg/kg, cyclophosphamide 80 mg/kg, fludarabine 100 mg/m2 |
| 3 | SM-AHN (MDS) | 67 |  | busulfan 6.4 mg/kg, fludarabine 150 mg/m^2^, melphalan 140mg/m^2^ |
| 4 | SM-AHN (MDS) | 65 |  | fludarabine 150 mg/m^2^, melphalan 140 mg/m^2^ |
| 5 | SM-AHN (PMF) | 64 |  | fludarabine 150 mg/m^2^, melphalan 140 mg/m^2^ |
| 6 | SM-AML | 56 |  | amsacrine 400 mg/m^2^, cytarabine 8000 mg/m^2^, cyclophosphamide 120mg/kg, fludarabine 120mg/m^2^, TBI 4 Gy |
| 7 | SM-AML (AML) | 61 |  | fludarabine 150 mg/m^2^, melphalan 140 mg/m^2^ |
| 8 | SM-AHN (MDS/MPNu) | 63 |  | fludarabine 150 mg/m^2^, melphalan 140 mg/m^2^ |
| 9 | SM-AML (AML) | 59 |  | fludarabine 3.9 mg/kg, melphalan 150 mg/m^2^ |
| 10 | MCL | 46 |  | amsacrine 11.3 mg/kg, cytarabine 225.4 mg/kg,  cyclophosphamide 79.3 mg/kg, fludarabine 3.4 mg/kg, TBI 4 Gy |
| 11 | MCL-AHN (ET) | 67 |  | busulfan, fludarabine |
| 12 | MCL-AHN (MDS) | 62 |  | fludarabine 150 mg/m^2^, treosulfan 42 g/m^2^ |
| 13 | MCL | 46 |  | busulfan 5.28 mg/kg, cyclophosphamid 100 mg/kg, fludarabine 150 mg/m^2^, thiotepa 10 mg/kg |
| 14 | SM-AHN (Post-ET MF) | 60 |  | fludarabine 150mg/m^2^, treosulfan 36g/m^2^ |
| 15 | ASM | 66 |  | fludarabine 120mg/m^2^, TBI 8 Gy |
| 16 | SM-AHN (MDS) | 64 |  | fludarabin 90mg/m^2^, TBI 2 Gy |
| 17 | MCL | 39 |  | fludarabine 160mg/m^2^, thiotepa 20mg/kg, TBI 12 Gy |
| 18 | SM-AHN (CMML) | 58 |  | busulfan 16mg/kg, fludarabine 150mg/m^2^ |
| 19 | SM-AHN (CMML) | 58 |  | fludarabine 90mg/m^2^, TBI 2 Gy |
| 20 | ASM | 60 |  | busulphan 8mg/kg, fludarabine 120mg/m^2^ |
| 21 | MCL | 75 |  | fludarabine 90mg/m^2^, TBI 3 Gy |
| 22 | ASM | 38 |  | cyclophosphamide 120mg/kg, TBI 12 Gy |
| 23 | SM-AHN (CMML) | 65 |  | amsacrine 400mg/m^2^, cytarabine 8000mg/m^2^  fludarabine 120mg/m^2^, melphalan 100mg/m^2^ |
| 24 | SM-AHN (MM) | 36 |  | thiotepa 10mg/kg, busulfan 9,6mg/kg, post cyclophosphamide |
| 25 | ASM | 61 |  | fludarabine 150mg/m^2^, treosulfan 42g/m^2^ |
| 26 | SM-AML | 65 |  | fludarabine 150mg/m^2^, treosulfan 30g/m^2^ |
| 27 | MCL-AHN (MDS/MPNu) | 56 |  | fludarabine 150mg/m^2^, treosulfan 42g/m^2^ |
| 28 | SM-AML | 22 |  | busulfan 12,8 mg/kg, cyclophosphamide 120mg/kg |
| 29 | SM-AML | 68 |  | amsacrine 400mg/m^2^, cytarabine 8g/m2, busulfan 6,4mg/kg, fludarabine 180mg/m^2^ |
| 30 | SM-AHN (MDS/MPNu) | 56 |  | busulfan 12.8mg/kg, Cyclophosphamide 120mg/kg |
| 31 | SM-AHN (CMML) | 58 |  | busulfan (60mg x hr/L) (AUC) fludarabine 150mg/m^2^, thiotepa 10mg/kg |
| 32 | SM-AHN (MDS) | 74 |  | amsacrine 400 mg/m^2^, cytarabine 4000 mg/m^2^, fludarabine 120 mg/m^2^, treosulfan 36000 mg/m^2^ |
| 33 | SM-AHN (CML) | 58 |  | fludarabine 150 mg/m^2^, treosulfan 36 g/m^2^ |
| 34 | SM-AHN (CMML) | 54 |  | fludarabine 120 mg/m^2^, TBI 8 Gy |
| 35 | SM-AHN (MDS/MPNu) | 61 |  | busulfan 80 mg x hr/L (AUC), fludarabine 150 mg/m^2^ |
| 36 | SM-AHN (CMML) | 84 |  | busulfan 6,4mg/kg, fludarabine 150 mg/m^2^, thiotepa 10mg/kg |
| 37 | SM-AHN (CMML) | 59 |  | busulfan 9,6 mg/kg, thiotepa 10 mg/kg |
| 38 | SM-AHN (CMML) | 61 |  | busulphan 6,4 mg/kg, fludarabine 150 mg/m^2^,  thiotepa 10 mg/Kg |
| 39 | SM-AML | 55 |  | fludarabine 120 mg/m^2^, treosulfan 30 g/m^2^ |
| 40 | SM-AHN (MDS/MPNu) | 50 |  | busulfan, cyclophosphamide |
| 41 | SM-AHN (MDS/MPNu) | 53 |  | busulfan 8 mg/kg, fludarabine 180 mg/m^2^ |
| 42 | SM-AHN (MDS/MPNu) | 66 |  | Fludarabine 150 mg, treosulfan 58,8g |
| 43 | SM-AHN (MDS/MPNu) | 51 |  | NA |
| 44 | SM-AML | 45 |  | NA |
| 45 | SM-AHN (MDS/MPNu) | 47 |  | BCNU 300 mg/m^2^, fludarabine 120 mg/m^2^, melphalan 140 mg/m^2^ |
| 46 | SM-AML | 61 |  | cytarabine 4 g/m^2^, BCNU 300 mg/m^2^, fludarabine 120 mg/m^2^, melphalan 110 mg/m^2^, mitoxantrone 10 mg/m^2^ |
| 47 | SM-AHN (MDS/MPNu) | 47 |  | cyclophosphamide 120mg/kg, busulfan 12,8 mg/kg |
| 48 | SM-AML | 68 |  | fludarabine 60 mg/m^2^, melphalan 110 mg/m^2^, thiotepa 10 mg/m^2^ |
| 49 | SM-AHN (MDS/MPNu) | 68 |  | fludarabine 60 mg/m^2^, melphalan 110 mg/m^2^, thiotepa 10 mg/m^2^ |
| 50 | SM-AHN (MDS/MPNu) | 66 |  | fludarabine 90 mg/m^2^, melphalan 110 mg/m^2^, thiotepa 10 mg/m^2^ |
| 51 | MCL-AHN (MDS/MPNu) | 65 |  | fludarabine 45 mg/m^2^, thiotepa 5 mg/ms2, treosulfan 30mg/m^2^ |
| 52 | SM-AML | 61 |  | fludarabine 120mg/m^2^, melphalan 110mg/m^2^, thiotepa 10mg/m^2^ |
| 53 | SM-AHN (MDS/MPNu) | 31 |  | busulfan 16mg/kg, cyclophosphamide 120mg/kg |
| 54 | SM-AHN (MDS/MPNu) | 46 |  | busulfan 992mg, cyclophosphamide 9294mg |
| 55 | SM-AML | 58 |  | BCNU 520mg, fludarabine 208mg, melphalan 190mg |
| 56 | SM-AHN (MDS/MPNu) | 53 |  | fludarabine 120mg/m^2^, TBI 8 Gy |
| 57 | SM-AHN (HES/CEL) | 51 |  | fludarabine 150mg, cytarabine, amsacrine, busulfan 8x0.8mg, |
| 58 | SM-AHN (HES/CEL) | 44 |  | fludarabine 30mg/m^2^, cyclophosphamide 40mg/kg, TBI 4 Gy |
| 59 | MCL-AHN (MDS) | 49 |  | thiotepa 5mg/kg, busulfan 4x0,8mg/kg, cylcophosphamide 60mg/kg |
| 60 | ASM | 28 |  | fludarabine 30 mg/m^2^, cytarabine 2000 mg/m^2^, amsacrine 100 mg/m^2^, busulfan 0,8 mg/kg, cyclophosphamide 60mg/kg |
| 61 | SM-AHN (MDS/MPNu) | 65 |  | fludarabine 180 mg/m^2^, amsacrine, cytarabine, busulfan 8x0,8 mg/kg |
| 62 | SM-AML | 38 |  | fludarabine 55 mg, TBI 8 Gy |
| 63 | SM-AHN (MDS/MPNu) | 66 |  | fludarabine 20 mg/m^2^, TBI 8 Gy |
| 64 | SM-AHN (HES/CEL) | 52 |  | fludarabine 30 mg/m^2^, TBI 8 Gy |
| 65 | SM-AHN (CMML) | 65 |  | fludarabin 63 mg, melphalan 294 mg, thiotepa 450 mg |
| 66 | SM-AHN (CMML) | 50 |  | fludarabine, treosulfan |
| 67 | ASM | 71 |  | Fludarabine 180 mg/m^2^, busulfan 6,4 mg/kg |
| 68 | MCL | 65 |  | fludarabine 30 mg/m^2^, treosulfan 12 g/m^2^, melphalan 100 mg/m^2^ |
| 69 | SM-AHN (CMML) | 66 |  | NA |
| 70 | SM-AML | 60 |  | Melphalan 100 mg/m², fludarabine 30 mg/m², TBI 8 Gy |
| 71 | MCL-AHN (HES/CEL) | 28 |  | Melphalan 100 mg/m², fludarabine 120 mg/m², TBI 8 Gy |
| Abbreviations: alloHCT, allogeneic stem cell transplantation; AHN, associated hematologic neoplasm; AML, acute myeloid leukemia; ASM, aggressive systemic mastocytosis; CMML, chronic myelomonocytic leukemia; Dx, diagnosis; ET, essential thrombocythemia; HES/CEL, hypereosinophilic syndrome/chronic eosinophilic leukemia; MCL, mast cell leukemia; MCL-AHN, mast cell leukemia with an associated hematologic neoplasm; MDS/MPNu, myelodysplastic/myeloproliferative neoplasm, unclassified; PMF, primary myelofibrosis; Post-ET MF, post-essential thrombocythemia myelofibrosis; r/r, refractory/relapse; SM, systemic mastocytosis; SM-AHN, systemic mastocytosis with an associated hematologic neoplasm  ^a^Dosage was not (fully) available in all patients. | | | | |

| **Table S6. Total body irradiation in 17 patients** | | | | | |
| --- | --- | --- | --- | --- | --- |
| **Total body irradiation** | **2 Gy** | **3 Gy** | **4 Gy** | **8 Gy** | **12 Gy** |
| Number of patients | 2 | 1 | 3 | 8 | 3 |
| Age in years at alloHCT; median (range) | 61 (58-64) | 75 | 46 (44-56) | 54 (28-66) | 39 (27-39) |
|  |  |  |  |  |  |
| **Diagnosis**  ASM, *n* (%)  SM-AHN, *n* (%)  SM-AML, *n* (%)  MCL±AHN, *n* (%) | -  2 (100)  -  - | -  -  -  1 | -  1 (33)  1 (33)  1 (33) | 1 (13)  3 (37.5)  3 (37.5)  1 (13) | 1 (33)  1 (33)  -  1 (33) |
|  |  |  |  |  |  |
| **Response before alloHCT**  Response, *n* (%)  Refractory/Relapse, *n* (%)  Upfront alloHCT, *n* (%)  Not available, *n* (%) | 1 (50)  -  1 (50)  - | -  1  -  - | -  3 (100)  -  - | 5 (62.5)  2 (25)  -  1 (12.5) | 1 (33)  2 (66)  -  - |
|  |  |  |  |  |  |
| **Conditioning**  Reduced intensity, *n* (%)  Myeloablative, *n* (%) | 2 (100)  - | 1  - | 2 (67)  1 (33) | -  8 (100) | -  3 (100) |
| Abbreviations: alloHCT, allogeneic stem cell transplantation; ASM, aggressive systemic mastocytosis; MCL±AHN, mast cell leukemia with/without an associated hematologic neoplasm; SM-AHN, systemic mastocytosis with an associated hematologic neoplasm; SM-AML, systemic mastocytosis with an acute myeloid leukemia | | | | | |

| **Table S7: Competing risk analysis of relapse and non-relapse mortality** | | | | | | | | |
| --- | --- | --- | --- | --- | --- | --- | --- | --- |
| **Characteristics** | | | **n** | **n (events)** | **Year 1** | **Year 3** | **Year 5** | ***P***^a^ |
| Disease mortality | | | | | | | | |
|  | Diagnosis | | 70 | 16 |  |  |  | 0.4 |
|  |  | ASM/SM-AHN | 29 | 6 | 11% (3%, 26%) | 16% (5%, 34%) | 23% (7%, 43%) |  |
|  |  | SM-AML | 28 | 5 | 11% (3%, 25%) | 14% (4%, 30%) | 14% (4%, 30%) |  |
|  |  | MCL±AHN | 13 | 5 | 34% (9%, 61%) | 44% (13%, 72%) | 44% (13%, 72%) |  |
| Non relapse mortality | | | | | | | | |
|  | Diagnosis | | 70 | 22 |  |  |  | 0.2 |
|  |  | ASM/SM-AHN | 29 | 6 | 10% (3%, 25%) | 19% (7%, 36%) | 25% (9%, 45%) |  |
|  |  | SM-AML | 28 | 11 | 36% (19%, 53%) | 40% (22%, 58%) | 40% (22%, 58%) |  |
|  |  | MCL±AHN | 13 | 5 | 23% (5%, 48%) | 34% (9%, 62%) | 45% (12%, 74%) |  |
| Abbreviations: ASM, aggressive systemic mastocytosis; MCL±AHN, mast cell leukemia with/without an associated hematologic neoplasm; SM-AHN, systemic mastocytosis with an associated hematologic neoplasm  ^a^ Gray’s test | | | | | | | | |

| **Table S8: Transplant characteristics in different AdvSM subtypes across different time periods** | | | | | | | |
| --- | --- | --- | --- | --- | --- | --- | --- |
|  | | | |  | **1999-2009** | **2010-2021** |  |
| Number of patients, *n* (%) | | | |  | 8 (11) | 63 (89) |  |
|  |  | | |  |  |  |  |
| **Diagnosis** | | | |  |  |  |  |
|  | ASM/SM-AHN, *n* (%) | | |  | 6/8 (75) | 24/63 (38) |  |
|  | SM-AML, *n* (%) | | |  | 2/8 (25) | 26/63 (41) |  |
|  | MCL±AHN, *n* (%) | | |  | 0/0 (0) | 13/63 (21) |  |
|  | Time from AdvSM diagnosis to alloHCT, median (range) | | |  | 1.4 (0.4-15.0) | 1.2 (0.0-16.7) |  |
|  |  | | |  |  |  |  |
| **Transplant** | | | |  |  |  |  |
|  | Graft source | | |  |  |  |  |
|  |  | PBSC, *n* (%) | |  | 7/8 (87) | 60/63 (96) |  |
|  |  | BM, *n* (%) | |  | 1/8 (13) | 3/63 (4) |  |
|  | Conditioning | | |  |  |  |  |
|  |  | Myeloablative, *n* (%) | |  | 4/7 (57) | 39/62 (63) |  |
|  |  | Reduced intensity, *n* (%) | |  | 3/7 (43) | 23/62 (37) |  |
|  |  | Total body irradiation, *n* (%) | |  | 3/8 (38) | 14/62 (23) |  |
|  |  |  | ≥8Gy, *n* (%) |  | 1/3 (33) | 10/14 (71) |  |
|  | Donor | | |  |  |  |  |
|  |  | MUD, *n* (%) | |  | 3/8 (38) | 22/54 (41) |  |
|  |  | MMUD, *n* (%) | |  | 3/8 (38) | 9/54 (17) |  |
|  |  | MRD, *n* (%) | |  | 2/8 (25) | 15/54 (28) |  |
|  |  | MMRD, *n* (%) | |  | 0/8 (0) | 1/54 (2) |  |
|  |  | Haploidentical, *n* (%) | |  | 0/8 (0) | 7/54 (13) |  |
|  |  | Recipient-donor sex mismatched, *n* (%) | |  | 3/8 (38) | 24/58 (41) |  |
|  |  | Donor age in years at alloHCT, median (range) | |  | 44 (43-45) | 33 (17-63) |  |
| Abbreviations: AHN, associated hematologic neoplasm; alloHCT, allogeneic hematopoietic stem-cell transplantation; AML, acute myeloid leukemia; ASM, aggressive systemic mastocytosis; BM, bone marrow; MCL±AHN, mast cell leukemia with/without an associated hematologic neoplasm; MMRD, HLA-mismatched related donor; MMUD, HLA-mismatched unrelated donors; MUD, HLA-matched unrelated donor; MRD, HLA-matched related donor; PB, peripheral blood; PBSC, peripheral blood stem cell; SM-AHN, systemic mastocytosis with an associated hematologic neoplasm | | | | | | | |
